# Development of a municipality index of environmental pressure in Campania, Italy

**DOI:** 10.2144/fsoa-2021-0055

**Published:** 2021-06-04

**Authors:** Antonio Pizzolante, Federico Nicodemo, Andrea Pierri, Amedeo Ferro, Biancamaria Pierri, Carlo Buonerba, Eleonora Beccaloni, Stefano Albanese, Bruno Basso, Pellegrino Cerino

**Affiliations:** 1 Centro di Referenza Nazionale per l’Analisi e Studio di Correlazione tra Ambiente, Animale e Uomo, Istituto Zooprofilattico Sperimentale del Mezzogiorno, Portici, 80055, Italy; 2 Dipartimento di Ambiente e Connessa Prevenzione Primaria, Istituto Superiore di Sanità, Rome, 00161, Italy; 3 Department of Earth Sciences, Environment & Resources, University of Naples Federico II, Naples, 80125, Italy; 4 Department of Earth & Environmental Sciences, Michigan State University, East Lansing, MI 48824, USA

**Keywords:** biomonitoring, environmental monitoring, Land of Fires, SPES trial

## Abstract

The Experimental Zooprophylactic Institute of Southern Italy (Istitituto Zooprofilattico Sperimentale del Mezzogiorno, IZSM) is a public health institution operating within the Italian National Health Service. Over the past 5 years [IZSM] has promoted several research studies and interventions in an effort to tackle the ‘Land of Fires’ phenomenon, caused by the continued trafficking and uncontrolled incineration of waste that has affected some areas of Campania for decades. In this article, a mathematical model that generates a municipality index of environmental pressure is presented. The model was developed by a multidisciplinary team led by an environmental engineer and included researchers in the fields of veterinary and human medicine, biology and computer science. This model may serve as a geostratification tool useful for the design of human biomonitoring studies, although it may also be employed for strategic planning of remediation programs and public health interventions.

## Lay abstract

The complex environmental scenario of the ‘Land of Fires’ phenomenon is caused by the continued trafficking and uncontrolled incineration of waste, and has affected some areas of Campania in Italy for decades. In this article a mathematical model that generates a municipality index of environmental pressure is presented. This index may be used for the design of biomonitoring studies as well as for planning of remediation interventions.

Since the 1980s, organized crime has been responsible for the continued illegal trafficking of industrial waste and toxic materials in the so-called ‘Land of Fires’ (Terra dei Fuochi [TdF]), a territory mostly located in the provinces of Naples and Caserta in the Campania region of southern Italy. The term ‘Terra dei Fuochi’ was introduced by the Italian environmental association Legambiente and refers to the fact that waste was abandoned and illicitly disposed through uncontrolled combustion [1].

In the early 1990s the Campania region suffered from a prolonged ‘waste crisis’ that lasted roughly 15 years and was caused by the inability of institutions to provide for the proper management of urban solid waste. Waste that accumulated in municipal areas was often set on fire by citizens exasperated by the nauseating smell [2], which generated fears of being exposed to dioxins among indwelling citizens [3].

Public concern about the threats posed to human health by environmental contamination grew in 2004, when Mazza and Senior used the expression ‘Triangle of Death’ to indicate a geographical area contained within the municipalities of Acerra, Nola and Marigliano of the province of Naples [4]. The authors concluded that the area was characterized by an unexpectedly high incidence of some forms of malignant neoplasms, which they assumed was the result of exposure to toxic waste. While the report was extensively covered by the media, its methodological limits, highlighted by other researchers [5] were largely ignored [3].

While a growing body of scientific evidence suggested that citizens dwelling in the provinces of Naples and Caserta could be affected by an increased risk of death, cancer, cancer-related mortality and congenital abnormalities [6–8], with an etiogenic role played by both naturally occurring and anthropic factors [9–12], the ‘bad reputation’ of the TdF severely harmed the local economy over the years and especially in 2013 and 2014, due to widespread consumer fears that the food produced in the Campania region was contaminated. As an example, in 2014 revenues from one typical product of the Campania region, water-buffalo mozzarella cheese, dropped by approximately 57 million Euros [13]. In order to tackle the social, economic and environmental emergency situation, a ‘Terra dei Fuochi Working Group’ was established by Law 6 / 2014 [14]. In an area of 92 hectares assessed in the region, 21 were identified as unsuitable for agrifood production by the Working Group, although none of the agricultural products analyzed were found to be noncompliant with regulatory limits for toxic substances [15]. The activities carried out by the Working Group had merits, but also suffered from several weaknesses: soil was the only environmental matrix analyzed (no air or water samples were assessed), not all municipalities were included in the environmental monitoring plan and no human biomonitoring survey was conducted.

Although human biomonitoring studies play a key role in assessing the threats posed by environmental pollution, only a few such studies have been conducted in the Campania region [16,17]. In a territory as vast and densely populated as that of the Campania region, which presents a surface area of 13,590 km^2^ and has over 5.5 million inhabitants residing in 550 municipalities divided into five provinces [18,19], a systematic biomonitoring survey can be effectively carried out at a regional level if the recruitment plan is wisely designed.

In this original work, we constructed a mathematical model that computes a synthetic index of environmental pressure at a municipality level (Municipality Index of Environmental Pressure [MIEP]). We computed the MIEP for all municipalities of the Campania region and then used it as a geostratification tool for the recruitment plan of a human biomonitoring survey at a regional level [20].

## Materials & methods

### Development of the MIEP

The MIEP is defined based on a pairwise comparison process between variables (Table 1) to which scores of relative significance are assigned through a multicriteria approach based on the Analytic Hierarchy Process method [21,22]. With this approach it was possible to move from a qualitative to a quantitative assessment of environmental sensitivity and to establish at the municipal scale the value of each variable in terms of its contribution to MIEP, according to the semantic classification proposed by Saaty (Table 2) [23].

**Table 1.** Variables used as sources of contamination.

Sources of contamination A_i_		Data source	Ref.
A	Contaminated sites A1 Contaminated landfills pursuant to Leg. Decree 152/06 A2 Plots TdF 5, 4, 3 respectively pursuant to Leg. Decree No. 56 of 9 March 2015 and Leg. Decree No. 191 of 19 August 2015	ARPAC: regional remediation plan	[36]
B	Areas of particular interest E1 Sites of national interest E2 Sites of regional interest E3 Illegal landfills E4 Landfills awaiting investigation	ARPAC: regional remediation plan	[36]
C	Zoning C1 Land use (residential, industrial, agricultural) C2 Population density	Corine Land Cover 2012	[37]
D	Status of water bodies: water analysis C1 Surface water bodies C2 Groundwater bodies	ARPAC: qualitative monitoring of water bodies	[38]
E	Potential hazard: soil analysis		[39]
F	Illegal spills and fires	SMA Campania	[40]
G	Waste management plants	ARPAC: plants authorized for waste management	[41]
H	Plots TdF Decree class 2a, 2b and 3, 4 and 5 of Class a	ARPAC: regional remediation plan	[36]

ARPAC: Campania Regional Environmental Protection Agency; Leg.: Legislative; TdF: Terra dei Fuochi.

**Table 2.** Saaty semantic scale for the attribution of weights.

Values a_ij_	Interpretation
1	i and j are equally important
3	i is slightly more important than j
5	i is much more important than j
7	i is very much more important than j
9	i is extremely more important than j

**Figure 1.**
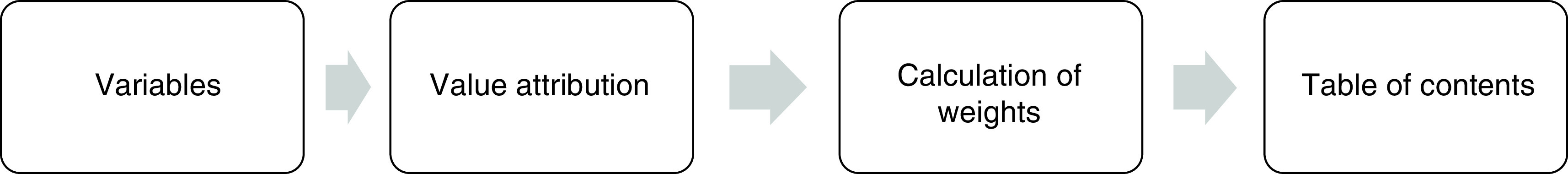
Algorithm for calculating the index.

Figure 1 shows a block diagram of the algorithm for calculating MIEP. In the process, A_i_ is the single source of contamination considered and a_ij_ is the numerical value resulting from the comparison between criteria i and j, which can vary from 1 to 9, where each value of the scale is assigned according to the criteria proposed in Table 2. In addition, intermediate values (e.g., 2, 4, 6, 8), not present in Table 2, were considered. The result of all the comparisons is reported in matrix A (Table 3). The latter was subsequently used to create the vector of the percentage weights (priority vector) of each single source taken into consideration (Table 1).

**Table 3.** Matrix of pairwise comparison between sources of contamination.

	A	B	C	D	E	F	G	H
A	1.00	3.00	2.00	3.00	3.00	5.00	7.00	9.00
B	0.33	1.00	3.00	2.00	2.00	4.00	6.00	8.00
C	0.50	0.33	1.00	3.00	4.00	4.00	6.00	6.00
D	0.33	0.50	0.33	1.00	1.00	3.00	5.00	6.00
E	0.33	0.50	0.25	1.00	1.00	3.00	5.00	6.00
F	0.20	0.25	0.25	0.33	0.33	1.00	5.00	6.00
G	0.14	0.17	0.17	0.20	0.20	0.20	1.00	3.00
H	0.11	0.13	0.17	0.17	0.17	0.17	0.33	1.00
Sum	2.95	5.88	4.17	10.70	11.70	20.37	35.33	45.00

Matrix A is an 8 × 8 square matrix in which the values resulting from pairwise comparisons are reported above the main diagonal, while the reciprocals of these values appear in the lower part. The a_ij_ values of matrix A have the following properties:

1.  If a_ij_ = a, then a_ji_ = 1/a, with a >0;
2.  If the variable A_i_ is judged to be of equal intensity relative to A_j_, then a_ij_ = a_ji_ = 1.

The last row in matrix A shows the sum of the individual elements that make up each column.

Matrix A was normalized, dividing each element a_ij_ by the sum relative to the j-th column. Subsequently, the average value of each i-th row of the matrix was calculated, defining the ‘priority vector’ as shown in Table 4.

**Table 4.** Matrix for construction of the ‘priority vector’.

	A	B	C	D	E	F	G	H	Priority vector
A	0.34	0.51	0.28	0.28	0.26	0.25	0.20	0.20	0.29
B	0.11	0.17	0.42	0.19	0.17	0.20	0.17	0.18	0.20
C	0.17	0.06	0.14	0.28	0.34	0.20	0.17	0.13	0.19
D	0.11	0.09	0.05	0.09	0.09	0.15	0.14	0.13	0.11
E	0.11	0.09	0.03	0.09	0.09	0.15	0.14	0.13	0.10
F	0.07	0.04	0.03	0.03	0.03	0.05	0.14	0.13	0.07
G	0.05	0.03	0.02	0.02	0.02	0.01	0.03	0.07	0.03
H	0.04	0.02	0.02	0.02	0.01	0.01	0.01	0.02	0.02

For each source of contamination A_i_, the model gave its percentage weight; in Table 5, the sources are sorted in descending order.

**Table 5.** Variables with their respective weights as a percentage.

Variable		Weight (%)
A	Contaminated sites	28.9
B	Areas of particular interest	20.0
C	Zoning	18.6
D	Status of water bodies: water analysis	10.6
E	Potential hazard: soil analysis	10.4
F	Illegal waste spills and fires	6.6
G	Waste management plants	3.0
H	Plots of land of the TdF Decree	1.9

TdF: Terra dei Fuochi.

To evaluate whether matrix A was consistent, or that the requirements of consistency and significance in the judgments expressed by the ‘preference indices’ were met, all the cells belonging to the i-th row of the non-normalized matrix were added together and multiplied vectorially by the sum of the priority vector and divided by the weight of the criterion relating to that row. In this way it was possible to quantify the consistency of each priority, as shown in Table 6.

**Table 6.** Substance matrix of variables used.

	A	B	C	D	E	F	G	H	Priority vector	Substance
A	0.34	0.51	0.28	0.28	0.26	0.25	0.20	0.20	0.29	9.02
B	0.11	0.17	0.42	0.19	0.17	0.20	0.17	0.18	0.20	9.33
C	0.17	0.06	0.14	0.28	0.34	0.20	0.17	0.13	0.19	9.09
D	0.11	0.09	0.05	0.09	0.09	0.15	0.14	0.13	0.11	8.81
E	0.11	0.09	0.03	0.09	0.09	0.15	0.14	0.13	0.10	8.78
F	0.07	0.04	0.03	0.03	0.03	0.05	0.14	0.13	0.07	8.39
G	0.05	0.03	0.02	0.02	0.02	0.01	0.03	0.07	0.03	8.24
H	0.04	0.02	0.02	0.02	0.01	0.01	0.01	0.02	0.02	8.60

The consistency index (CI) of the entire matrix A was calculated using the following relation, where *λ* represents the maximum eigenvalue of matrix A and *n* the dimension of the matrix itself (Equation 1):

(Eq. 1) $$CI=\frac{\left(\lambda_{\max}-n\right)}{\left(n-1\right)}$$

In Equation 1, if the value of CI is equal to 0 then the matrix is consistent; if it deviates from *n*, then the matrix is not perfectly consistent, although the methodology used accepts a low degree of inconsistency because this does not affect the validity of the result obtained. As a first approximation, the maximum eigenvalue of matrix A can be evaluated by referring to the average of the consistencies relating to the individual variables; the result is a maximum eigenvalue equal to 8.70, which is close to the dimension *n* of matrix A.

Once the CI was known, it was possible to define the random consistency index; for matrix A (with *n* = 8) the value of this index is equal to 1.41 (Table 7).

**Table 7.** Values of the RCI as a function of matrix order.

Matrix order	1	2	3	4	5	6	7	8	9	10	11	12	13	14	15
RCI	0.00	0.00	0.58	0.90	1.12	1.24	1.32	1.41	1.45	1.49	1.51	1.48	1.56	1.57	1.59

RCI: Random consistency index.

At this point it was possible to evaluate the consistency ratio (CR) of matrix A, defined by the following equation (Equation 2):

(Eq. 2) $$CR=\frac{CI}{RCI}$$

For matrix A to be consistent, the value of CR must be less than 0.1. In the specific case, Equation 2 gave a CR of 0.07, indicating the consistency of the matrix.

Once the ‘P_i_’ weights to be assigned to each pressure variable were determined, the MIEP values were determined. Specifically, for each municipality, MIEP was calculated by a linear combination of the set of pressure variables considered, multiplied in turn by specific amplification coefficients as functions of the number, type, extent, hazard, environmental status and impact of the variable itself. These coefficients were introduced in such a way as to be able to define the model on the environmental and territorial characteristics of each municipality in the Campania region. The MIEP relating to the i-th municipality of Campania region is expressed by the following relationship:

$$\begin{array}{c} MIEP_{i}^{}\;=\;MIEP{\;}_{i\left(contaminated\;sites\right)}+MIEP_{i\left(areas\;of\;interest\right)}+MIEP_{i\left(zoning\right)}+MIEP_{i(water)}+MIEP_{i\left(soil\right)}\\ MIEP_{i(illegal\;spills)}+MIEP_{i\left(waste\;managment\right)}+\;MIEP_{i\left(\;TdF\;plots\right)} \end{array}$$

In order to make a comparison between the environmental pressure indices determined, the variable was normalized in such a way as to have values between 0 and 100. The normalization operation was carried out through application of the following relationship:

$$MIEP_{i}^{norm}=\frac{MIEP_{i}^{}-MIEP_{\min}^{}}{MIEP_{\max}^{}-MIEP_{\min}^{}}\times 100$$

We use the abbreviation MIEP_i_ to refer to normalized MIEP_i_ from now on in text unless otherwise specified.

### Definition of model variables

#### ‘Contaminated sites’ variable

The ‘contaminated sites’ variable includes contaminated landfills as defined by Legislative Decree 152/2006 and plots of land of classes 3, 4 and 5 as indicated in Decree No. 56 of 9 March 2015 and No. 191 of 19 August 2015 relating to TdF. Following investigations by the Campania Regional Environmental Protection Agency (ARPAC), the plots of land defined by decree were further classified as shown in Table 8.

**Table 8.** Classes of agricultural use for plots of land of the Terra dei Fuochi (Land of Fires) Decree.

Agricultural use class	Definition
A	Land suitable for agrifood production
A1	Land suitable for agrifood production after removal of waste and analysis of sedimentation areas
B	Land with limitation for certain agrifood productions under certain conditions
NC	Nonclassifiable land
D	Land where agrifood production is prohibited

In determining the environmental pressure index, only types B, NC and D of class 3, 4 and 5 plots of land were considered. Table 9 shows the p′ scores assigned: the criterion adopted in this case was to attribute the highest significance, in terms of hazard, to contaminated landfills.

**Table 9.** p′ score by type of contaminated sites.

Contaminated sites	p′ score
TdF class 3, 4, 5 type B plots	3
TdF class 3, 4, 5 type NC plots	5
TdF class 3, 4, 5 type D plots	7
Contaminated landfills	9

TdF: Terra dei Fuochi.

A further p″ score was attributed according to the specific spatial extension of these sites, assuming that the degree of pressure is directly proportional to the extent of the site, as shown in Table 10.

**Table 10.** p″ score referred to extent of contaminated sites.

Contaminated sites surface area (m^2^)	p′′ score
0–2500	2
2500–5000	3
5000–10,000	4
10,000–20,000	5
20,000–50,000	7
>50,000	9

The pressure index relating to the variable in question is expressed by the following mathematical relationship, where the sum is extended to all the contaminated sites present in a municipality:

$$MIEP_{\left(contaminated\;sites\right)}=\sum_{i=1}^{n}p_{i}^{“}\cdot p_{i}^{”“}$$

#### ‘Areas of particular interest’ variable

This variable includes sites of national and regional interest present in the area, illegal landfills awaiting investigation, as well as potentially contaminated sites investigated. For each area of interest considered, a preliminary score p′ is assigned based on the different degrees of presumed pressure. In the specific case, sites of national interest are assigned the highest score, followed by illegal landfills, areas awaiting characterization and, finally, potentially contaminated sites. The potential risk index relating to the ‘areas of particular interest’ variable was evaluated by assigning the score to the single element of the variable, as shown in Table 11. From an analytical point of view, the environmental pressure index for the considered variable is expressed by the following relationship:

$$MIEP_{\left(areas\;of\;interest\right)}=\sum_{i=1}^{n}p_{i}^{”}$$

**Table 11.** p′ scores for the ‘areas of particular interest’ variable.

Areas of particular interest	Score
Sites of national interest	9
Illegal landfills	7
Areas awaiting characterization	5
Potentially contaminated sites	3

#### ‘Zoning’ variable

The ‘zoning’ variable considers the different impacts, both direct and indirect, exerted by different land uses (urban, agricultural and commercial/industrial areas). To define the environmental pressure index relating to the ‘zoning’ variable, scores (p′) were assigned to the different land use destinations present in the 2012 edition of the ‘Corine Land Cover’ map, based on qualitative assessments of pressures exerted in each of them. Based on the available data, the criteria followed consisted of attributing a greater weight, in terms of environmental hazard, to residential areas, followed by industrial areas and then agricultural and wooded areas (Table 12).

**Table 12.** Scores attributed to types of land use.

Type of residential use	Score
Wooded	0
Residential 1	1
Residential 2	2
Agriculture	3
Residential 3	4
Residential 4	5
Industrial	7
Residential 5	8
Residential 6	9

To represent the relative extension of each intended use over the entire municipal area, a specific p′′ indicator was introduced, divided into the following parameters:

1.  Residential area/municipal area
2.  Industrial area/municipal area
3.  Wooded area/municipal area
4.  Agricultural area/municipal area

Tables 13 & 14 show the bands considered and the relative scores assigned.

**Table 13.** Scores assigned to the p″ indicator for industrial and residential areas.

Industrial area/municipal area (%)	Score	Residential area/municipal area (%)	Score
0–1	1	0–7	1
1–4	3	7–12	3
4–10	5	12–33	5
10–21	7	33–56	7
21–100	9	56–100	9

**Table 14.** Scores assigned the to p″ indicator for agricultural and wooded areas.

Agricultural area/municipal area (%)	Score	Wooded area/municipal area (%)	Score
0–24	1	0–10	1
24–43	3	10–25	3
43–60	5	25–47	5
60–75	7	47–78	7
75–100	9	78–100	9

The environmental pressure index associated with the zoning variable is expressed analytically by the following expression:

$$MIEP_{\left(zoning\right)}=\sum_{i=1}^{n}p_{i}^{“}\cdot p_{i}^{”“}$$

where the sum is extended to the n land uses present in a municipality.

#### ‘Illegal waste spills & fires’ variable

The ‘illegal waste spills and fires’ variable indicates the presence of abandoned waste and uncontrolled fires. To determine the pressure index relating to this variable, the number of waste spills detected in the municipalities by the monitoring activity carried out by SMA Campania (https://www.smacampania.info/chi-siamo/) was taken into consideration. The environmental pressure index relating to the ‘illegal waste spills and fires’ variable coincides with the number of spills detected on a municipal basis. It can be mathematically formalized by the following formula:

$$MIEP_{\left(illegal\;spills\right)}=\sum_{i=1}^{n}i$$

#### ‘Waste management plants’ variable

This variable includes incineration, storage, composting, selection, purification, recovery, scrapping plants and controlled landfills. A p′ score is assigned to the specific type of plant. Table 15 shows the types of plants considered and the relative scores assigned.

**Table 15.** p′ score for each waste treatment plant type identified.

Treatment plant type	Score
Controlled landfill	3
Scrapping plant	3
Other	3
Recovery	3
Purification	6
Selection/sorting	6
Composting	6
Storage	7
Incineration	9

In assigning the weights, it was decided to attribute the same significance to the types of plants which, on the basis of the waste treated, present the same level of environmental hazard. The environmental pressure index relating to the variable was mathematically formalized by the following relationship:

$$MIEP_{\left(waste\;management\right)}=\sum_{i=1}^{n}p_{i}^{‘}$$

#### ‘TdF plots of land decree’ variable

As regards the plots of land of the TdF Decree, defined in the Directive of 23 December 2013, all the plots for which a site-specific investigation has not yet been carried out (2A and 2B) and those of classes 5, 4 and 3 which, following investigations, are not contaminated (Table 16) were taken into consideration.

**Table 16.** Scores attributed to plots of the TdF decree.

Decree plots of land	Score
Class 2A	9
Class 2B	7
Class 3, 4, 5A	5

The environmental pressure index relating to the variable ‘TdF decree plots’ was assessed by assigning the score p′ to the single element of the variable according to the formula presented below.

$$MIEP_{\left(TdF\;plots\right)}=\sum_{i=1}^{n}p_{i}^{’}$$

#### ‘Potential hazard: soil analysis’ variable

The potential hazard variable was created starting from analysis of the spatial distribution of the concentration values of contaminants using spatial statistics models, which made it possible to reconstruct continuous concentration areas on the entire regional territory and to estimate the probability of exceeding the legal limits or reference values in areas not covered by sampling [24–26]. The ‘potential hazard’ map is very useful, insofar as, in addition to enabling the identification of areas potentially at risk, it serves to define the background/baseline values of the various geochemical elements investigated, according to the various types present in the substrate. On the basis of this cartography, the indicator ‘potential hazard area/municipal area’ was taken into consideration for each municipality. The values of this ratio were divided into five classes defined on the basis of a classification of a ‘natural breaks’ type [27]. A p′ score was applied to each of the intervals thus defined in Table 17.

**Table 17.** p′ scores for relationships identified between CTC and municipal area.

Surface area exceedances CTC/municipal area (%)	Score
0–9	0
9–30	3
30–55	5
55–80	7
80–100	9

CTC: Contamination threshold concentration.

The environmental pressure index relating to this variable is determined through the relationship shown below, where *a* represents the number of analytes the concentrations of which have exceeded regulatory limits and the sum present is extended to the *n* areas potentially at risk, with reference to the municipal territory:

$$MIEP_{\left(soil\right)}=a\sum_{i=1}^{n}{P^{‘}}_{i}$$

#### ‘Water bodies status: water analysis’ variable

This variable takes into account the quality status of groundwater bodies. To fully define the environmental pressure index relating to this variable, a series of attributes were introduced that indicate the qualitative status (the ‘chemical status of groundwater’ index) and a series of indicators that take into account the percentage municipal coverage of the underground aquifer. In fact, the chemical status of groundwater index summarizes the qualitative state of groundwater based on a comparison of the average annual concentrations of the chemical parameters analyzed with the relative quality standards and threshold values defined at national level by Legislative Decree 30/09 [28], also taking into account natural background values. Based on this, a p′ score was assigned to the qualitative status of the groundwater body. The highest score was assigned to the ‘poor’ status, insofar as this condition presupposes exceeding of the reference values (standard and threshold), even for a single parameter. The assigned score took into consideration the anthropogenic or natural origin of the aforementioned exceedances. Table 18 shows the scores assigned.

**Table 18.** p′ scores attributed to qualitative status of groundwater bodies.

Qualitative status of groundwater bodies	Score
Good	0
Particularly good	1
Not monitored	3
Poor	9

Subsequently, for each municipality, the indicator ‘groundwater body area/municipal area’ was introduced to take into account the extension of the groundwater body in relation to the municipal area.

The values of these indicators were divided into a series of intervals (bands), established according to a classification of the ‘natural breaks’ type. A second p″ score was then assigned to each interval thus defined. Table 19 shows the bands considered and the relative scores assigned. The environmental pressure index related to the variable was evaluated through the following relationship:

$$MIEP_{\left(water\right)}=\sum_{i=1}^{n}p_{i}^{’}\cdot p_{i}^{‘’}$$

**Table 19.** p″ score attributed to ratio of areas.

Groundwater surface area/municipal surface area (%)	Score
0–15	1
15–37	3
37–60	5
60–85	7
85–100	9

where the sum is extended to all groundwater bodies within a municipality.

### Design of a regional-scale human biomonitoring study

Impact areas are made up of an aggregation of municipalities, chosen in an arbitrary manner according to criteria of spatial contiguity and technical/logistical needs. The Impact Area Pressure Index is calculated as the average of the municipalities that make it up, weighted with respect to the resident municipal population [29]. Relative to the Impact Area Pressure Index, impact areas are classified as high, medium and low impact for values ≥50, <50 but >25, and <25, respectively. Within the areas, clusters are identified consisting of subaggregations of municipalities grouped according to the MIEP following the Jenks natural breaks classification [27]. Municipalities that fall into particular geographical contexts in which there is a limited source of pollution can nevertheless be aggregated into specific clusters.

## Results

### Calculation of the environmental pressure index on a municipal basis

Following the application of multicriteria analysis, the contaminated sites variable assumes greater significance than the others considered, because health risk has been ascertained (exceeding risk threshold concentrations) for the potentially exposed population. With a difference of about 9%, it follows the ‘areas of particular interest’ variable, which includes all those territorial circumstances in which there has been an exceeding of contamination threshold concentrations in one or more environmental compartments investigated through sampling and analytical tests, thus denoting phenomena of potential contamination in progress. This is followed on a par by the ‘zoning’ variable, which directly considers the different land uses (residential areas, industrial areas, agricultural areas etc.), with particular reference to the set of activities present and potential pressures exercised on environmental sectors. ‘Status of water bodies’ and ‘potential hazard’ come next, with a difference of about 8%; these two variables indicate the degree of pressure determined by the quality status of the underground/surface water bodies that pass through and by the presence of soil contamination phenomena, also attributable to natural factors.

**Figure 2.**
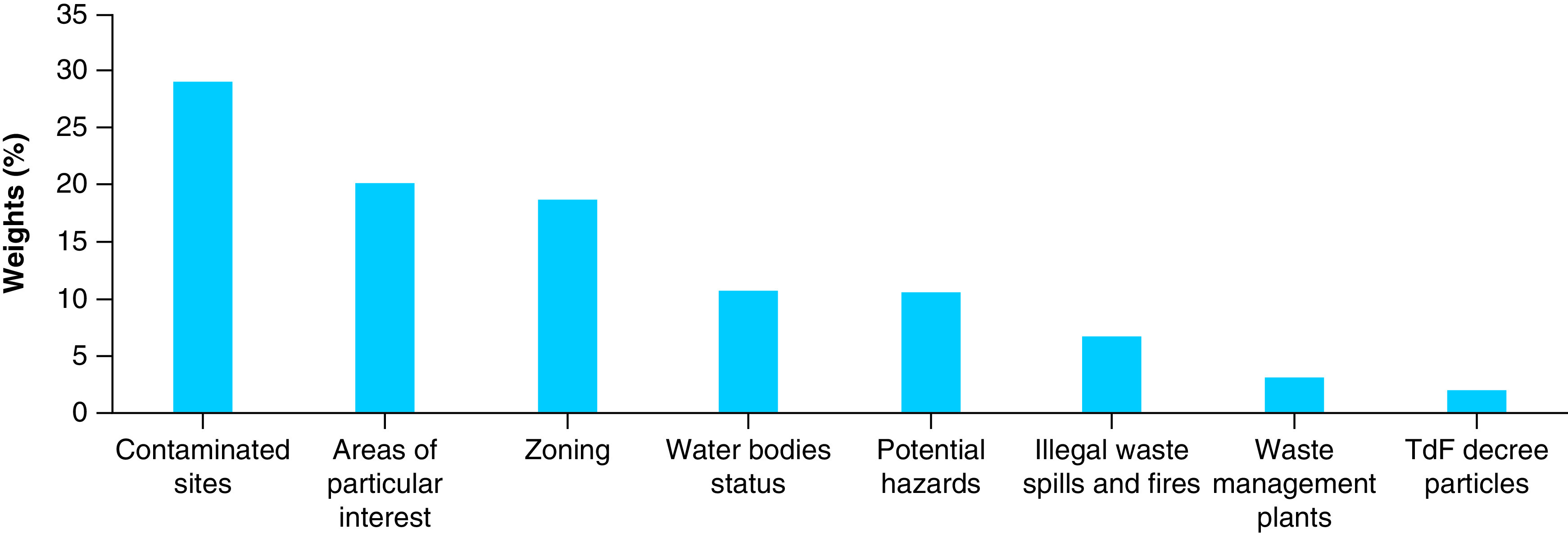
Percentage weights of variables. TdF: Terra dei Fuochi.

The ‘illegal waste spills and fires’ variable was considered more important than the last two variables (waste management plants and plots of land of the TdF Decree) because the former considers mainly authorized plants with controlled management, while the latter considers the TdF plots for which a site-specific survey has not yet been carried out (classes 2A and 2B) and those which, following the investigations, are not polluted. Figure 2 shows a bar graph with the attribution of percentage scores for each single variable entered.

**Figure 3.**
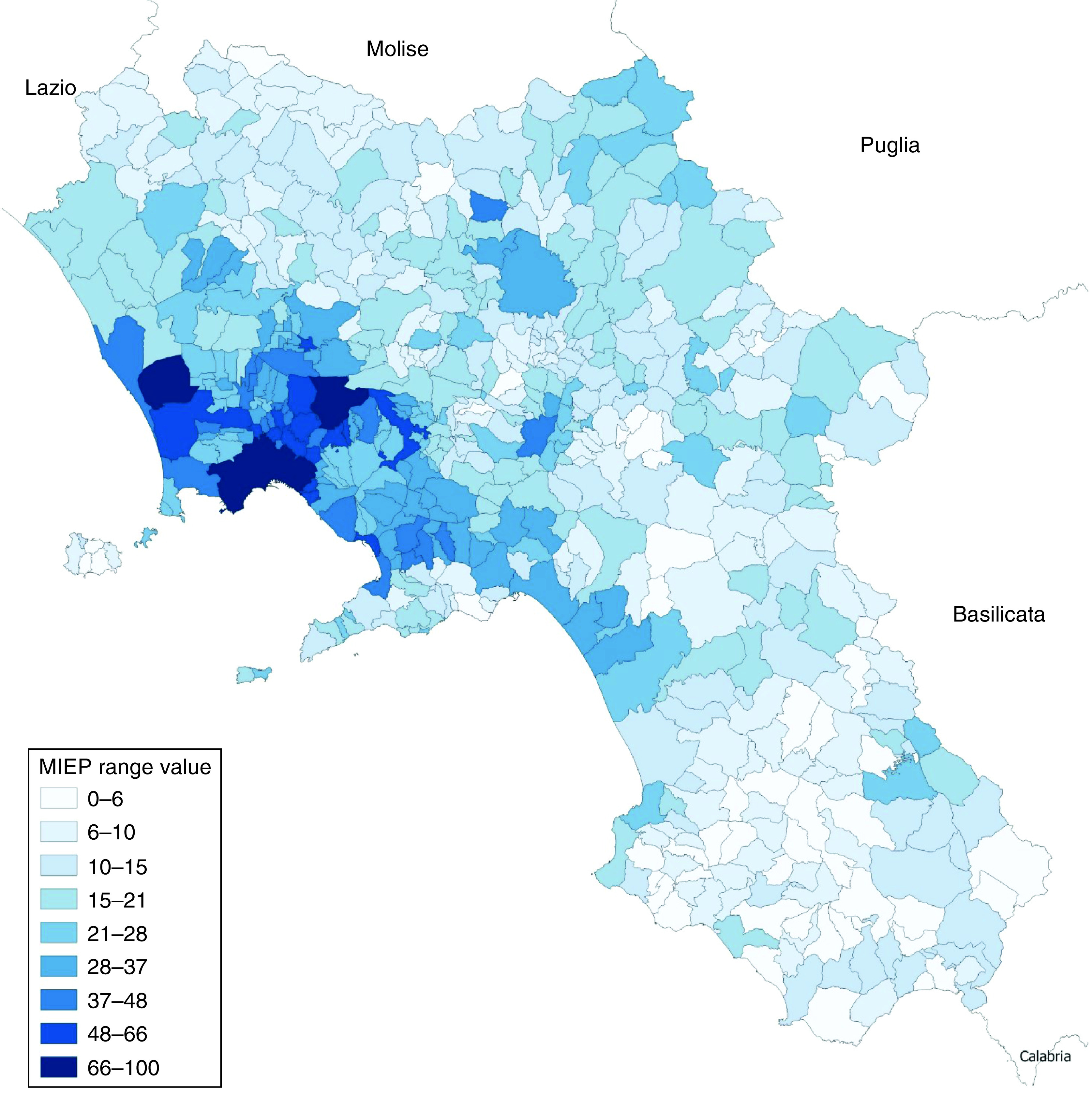
Representation of Municipality Index of Environmental Pressure.

For each municipality in the Campania region, the model gave an MIEP value ranging from 0 to 100 (Supplementary Table 1 & Figure 3).

Upon analyzing the results, it is observed that the municipalities with the highest MIEP are concentrated mainly in the provinces of Naples and Caserta, areas known for the massive presence of specific and/or widespread sources of pressure and, at the same time, subject to frequent monitoring and environmental investigation which allow a more meaningful analysis to be developed. In particular, high values of the index are found in all the municipalities that are part of Litorale Agro-Domitio, the metropolitan area of the Municipality of Naples, the Vesuvian hinterland and Ager Nolanus. Other sensitive areas coincide with Agro-Nocerino Sarnese, Valle del Sabato and some municipalities of Piana del Sele, although to a lesser extent.

### Design of a human biomonitoring study in the Campania region

**Figure 4.**
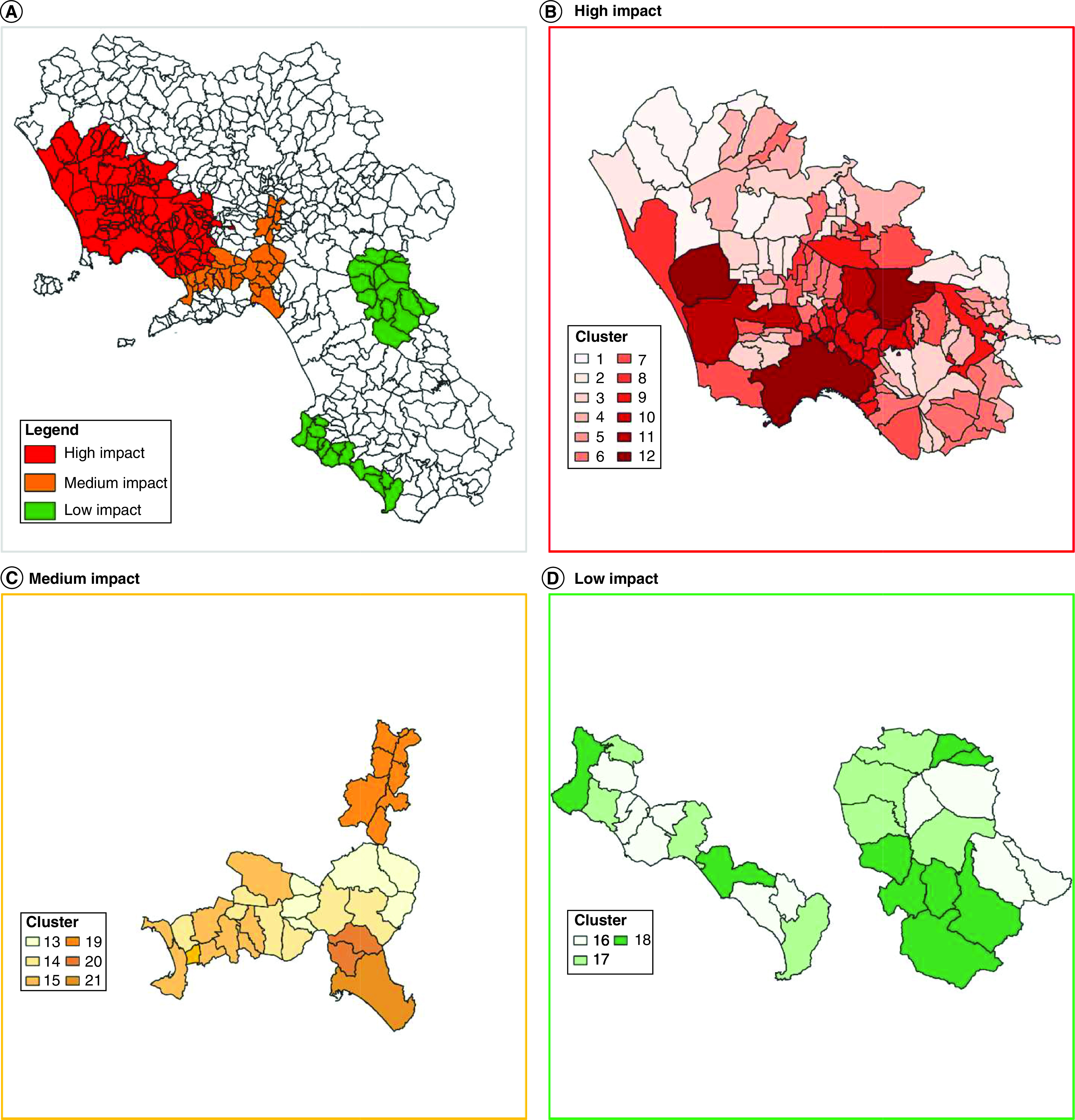
Identification of impact areas and clusters for the design of a biomonitoring study in the Campania Region.

For the design of a human biomonitoring study to be conducted at a regional level, a total of 174 municipalities of the Campania region, representing 80% of the regional population, were chosen on the basis of geographical contiguity and logistical constraints. First, the municipalities were grouped into three areas (Table 20 & Supplementary Table 2) based on geographical contiguity, and were classified at high, medium and low environmental pressure (Figure 4) based on the arithmetic mean MIEP weighted for the total number of municipality residents. We then grouped municipalities within the same area into ‘clusters’, which represents the actual tool for geostratification to be used for the biomonitoring study, following the ‘natural breaks’ approach [27], with the exception of municipalities of the Sabato and Irno Valleys, which were included into three separate clusters (Valle dell’Irno 1, Valle del Sabato 1 and Valle dell’Irno 2), because of peculiar local sources of contamination, the effect of which our model was not designed to capture [30,31].

**Table 20.** Identification of impact areas applicable to the geostratified recruitment plans of a biomonitoring study of the Campania region population.

Impact area description	Municipalities (n)	Resident population (2011 census)	Pressure index weighted on resident population
Most of the corresponding provinces of Naples and Caserta, located in the Voltuno-Regi Lagni plain, Campi Flegrei and Vesuvian municipalities	114	3,405,056	57.5
Area south of the province of Naples, north-west of the province of Salerno and west of the province of Avellino, located in the plain of the Sarno river and Solofra-Cavaiola, in Valle dell’Irno and in Valle del Sabato	32	765,513	35.8
Municipalities located in the south-west and north-east of the province of Salerno, located along the Cilento coast and in the innermost part of Valle del Sele-Tanagro	28	76,427	13.0

## Discussion

Existing literature provides several examples of synthetic environmental pressure/risk indices, sometimes for purposes that differ substantially from those pursued by our research group. Vacca *et al.* applied a model of contamination risk analysis in the industrial district of Ottana (Nuoro, Italy), an area characterized by the massive presence of chemical and textile industries which have strongly modified the entire territorial and social structure since the early 1970s [32]. In this study, carried out within a programmed agreement between the Provincial Administration of Nuoro and the Department of Botany at the University of Sassari, the authors evaluated microdischarges in the floodplain area of the Tirso river within the industrial district of Ottana, with the aid of geographic information system (GIS) applications, in order to correlate the shapes, dimensions, typology and toxicity of the materials contained therein with the characteristics and quality of the soil. A total of 28 sites were identified and subjected to a relative risk assessment; these sites were contaminated by materials of various origins and nature (drums containing support materials for chemical production, furnishings, tanning and meat processing residues, abandoned automobiles, animal carcasses, plastics and tires, and non-inert and often highly fragmented asbestos). The relative risk analysis model applied by Vacca *et al.* is based on a score and weight system which takes into account 24 analysis factors, grouped into three main categories: characteristics of the waste, migration routes of contaminants and typology of receptors. Each factor is measured by an index score with a range of variability from 0 to 10 proportional to the incidence of the factor itself on the risk analysis. The score obtained is then multiplied by a weight (Pesoi), which varies from 1 to 3 depending on the significance of the factor’s contribution to overall risk conditions. In accordance with the risk indices obtained, three priority areas were identified (low, medium and high), in a manner similar to the methodology we used.

In the study by Chrysochoou *et al.*, the authors present a risk assessment model applied to a large number of brownfield sites in large areas (municipalities, counties, states or other types of districts), which is useful for planning reclamation and redevelopment actions [33]. The model uses socioeconomic aspects and sustainable growth and the environment, for each of which the authors propose a synthetic index calculated on the basis of territorial variables. Socioeconomic variables include population density, property values and unemployment rates, which collectively indicate how brownfield regeneration can contribute to economic growth. The environmental index incorporates variables that represent the potential source of contamination, routes of exposure and the presence of targets. The application of this model to the town of New Haven, Connecticut led the authors to identify four areas for intervention out of the 47 analyzed.

In the study by Martuzzi *et al.*, which evaluated the impact on public health of the waste emergency in the provinces of Naples and Caserta, the authors developed a municipal index of environmental pressure from waste disposal, which was used in the analysis of geographical correlation with epidemiological data [6]. Starting with a census of waste treatment plants and their characteristics in the study area, the authors assigned a hazard to each site and to the impact area within a 1-km radius of the identified site. The impact areas and the corresponding hazard levels were reaggregated at the municipal level to derive a municipal hazard index as well as a municipal index of pressure from waste disposal, which considered the surface area and the population in each impact area. In the geographical correlation study, a discretized index was used in five increasing risk classes; disaggregation of distribution of the risk index was carried out following two different methodologies, including ‘adjusted’ quintiles and natural breaks, as was ours. The natural breaks method was used in Martuzzi’s work to divide the environmental pressure index into five homogeneous classes; the groups of municipalities obtained are not of the same number, but remain internally homogeneous and non-homogeneous [6]. The group with the greatest environmental pressure from waste disposal covers eight municipalities: Acerra, Aversa, Bacoli, Caivano, Villa Literno, Castel Volturno, Giugliano in Campania and Marcianise. The authors believe that use of the results of this methodology in the analysis of geographical correlation with health data makes it possible to evaluate whether there is a relationship between the risk of mortality or congenital malformations and classes of municipalities at different levels of environmental pressure.

The Experimental Zooprophylactic Institute of Southern Italy, with headquarters in Portici, is one of the ten Zooprophylactic Institutes in Italy operating within the National Health Service on Hygiene and Veterinary Public Health as a technical and scientific tool for the State and of the Campania and Calabria regions. In response to the complex social, environmental and economic situation caused by the TdF phenomenon, the Experimental Zooprophylactic Institute of Southern Italy collaborated closely with the TdF working group, carrying out additional monitoring surveys both on food (QR Code Campania project [34]) and on the environment (Campania Trasparente project [35]). This model was developed in the context of the experience accumulated in the field of environmental and food monitoring, and represents an innovative tool aimed at increasing knowledge of the environmental context of the Campania region through an objective, integrated and organic synthesis of complex environmental phenomena and territorial dynamics. The particular context we are studying is characterized by the presence of specific and/or widespread sources of pressure, of different types and sizes, variously distributed over the territory and capable of generating highly heterogeneous impacts. The analysis assumes the municipal limits as a territorial reference because many of the environmental data taken into consideration, produced by different bodies (municipalities, provinces, ARPAC, Campania region, universities etc.), were aggregated on this basis. In this way, an attempt was made to safeguard the spatial detail of the data. Within the limits of this analysis, it is necessary to bear in mind the approximate and, in part, subjective component inherent in the attribution of scores of significance relative to the variables considered and linked to qualitative assessments of the potential impacts generated, on any transport mechanisms of active contaminants and on potentially exposed targets (food, humans etc.). The strengths of this approach lie in the wide set of variables considered which, to varying degrees, contribute to determining the ‘environmental balance’ of the municipality assessed. The proposed model enables the simultaneous evaluation of a large number of variables and the objective expression of the environmental pressure relating to the municipal territory, and summarizes it in a single index. When applied as part of the design of a monitoring study on a large number of municipalities, this index allows them to be grouped on the basis of similar environmental pressures, making it possible to identify a geostratification unit on which to perform population sampling, with significant resource savings and faster recruitment [20].

## Conclusion

The model proposed here is useful for the global and synthetic assessment of environmental pressure on a municipal basis. As shown, it can also be applied to aggregations of municipalities. Furthermore, it can be used in the context of institutional actions for the planning and monitoring of improvements on a local or regional scale. Finally, the proposed MIEP represents a basis for geostratification of the sample in the context of population biomonitoring studies on a regional scale, as in the described biomonitoring study design applicable to the Campania region.

## Future perspective

In the future, the threats posed by widespread pollution to human health are going to increase. In the peculiar context of the TdF, environmental contamination has not been the result of industrial activities, but of illegal activities. Little progress has been made in Campania regarding remedial interventions. Mathematical models capable of assessing the levels of pollution in a certain area represent a valuable tool to identify areas in need of remediation. Conversely, the need for biomonitoring studies is going to become more and more compelling, and the implications of biomarkers of exposure for the health of the individual citizen remain unknown. More studies are required to interpret the results of biomonitoring studies at an individual level.

**Summary points**

**Background**

- We constructed a mathematical model that computes a synthetic index of environmental pressure at a municipality level (the Municipality Index of Environmental Pressure [MIEP]).
- We computed the MIEP for all municipalities of the Campania region and used it as a geostratification tool for the recruitment plan of a human biomonitoring survey at a regional level.

**Methods**

- The MIEP is defined based on a pairwise comparison process between variables to which scores of relative significance are assigned through a multicriteria approach based on the analytic hierarchy process method.

**Results**

- For each municipality in the Campania region, the model gave a MIEP value ranging from 0 to 100.
- It was observed that the municipalities with the highest MIEPs are concentrated mainly in the provinces of Naples and Caserta.

**Discussion**

- The model proposed here is useful for the global and synthetic assessment of environmental pressure on a municipal basis.
- The model can also be applied to aggregations of municipalities.
- The model can be used in the context of institutional actions for the planning and monitoring of improvements on a local or regional scale.
- Finally, the proposed MIEP represents a basis for geostratification of the sample in the context of population biomonitoring studies on a regional scale, as in the described biomonitoring study design applicable to the Campania region.
