# Supplementary material for: Development of a municipality index of environmental pressure in Campania, Italy
Source: Future Sci OA. 2021 Jun 4;7(7):FSO720. doi: 10.2144/fsoa-2021-0055 (PMC8256331; doi:10.2144/fsoa-2021-0055)
Supplement: Supplementary file 1 [file fsoa-07-720-s1.docx]

| Municipality | MPI |
| --- | --- |
| Acerno | 5 |
| Acerra | 81 |
| Afragola | 53 |
| Agerola | 17 |
| Agropoli | 26 |
| Aiello del Sabato | 22 |
| Ailano | 16 |
| Airola | 25 |
| Albanella | 12 |
| Alfano | 5 |
| Alife | 13 |
| Altavilla Irpina | 17 |
| Altavilla Silentina | 12 |
| Alvignano | 14 |
| Amalfi | 16 |
| Amorosi | 12 |
| Anacapri | 19 |
| Andretta | 26 |
| Angri | 44 |
| Apice | 16 |
| Apollosa | 15 |
| Aquara | 9 |
| Aquilonia | 6 |
| Ariano Irpino | 17 |
| Arienzo | 9 |
| Arpaia | 7 |
| Arpaise | 9 |
| Arzano | 63 |
| Ascea | 17 |
| Athena Lucana | 24 |
| Atrani | 33 |
| Atripalda | 28 |
| Auletta | 6 |
| Avella | 20 |
| Avellino | 40 |
| Aversa | 38 |
| Bacoli | 27 |
| Bagnoli Irpino | 7 |
| Baia e Latina | 12 |
| Baiano | 11 |
| Barano d'Ischia | 4 |
| Baronissi | 23 |
| Baselice | 20 |
| Battipaglia | 32 |
| Bellizzi | 26 |
| Bellona | 14 |
| Bellosguardo | 9 |
| Benevento | 32 |
| Bisaccia | 17 |
| Bonea | 18 |
| Bonito | 12 |
| Boscoreale | 36 |
| Boscotrecase | 24 |
| Bracigliano | 20 |
| Brusciano | 31 |
| Bucciano | 10 |
| Buccino | 18 |
| Buonabitacolo | 15 |
| Buonalbergo | 12 |
| Caggiano | 9 |
| Caianello | 13 |
| Caiazzo | 13 |
| Cairano | 6 |
| Caivano | 65 |
| Calabritto | 9 |
| Calitri | 15 |
| Calvanico | 16 |
| Calvi | 21 |
| Calvi Risorta | 16 |
| Calvizzano | 26 |
| Camerota | 6 |
| Camigliano | 9 |
| Campagna | 9 |
| Campolattaro | 19 |
| Campoli del Monte Taburno | 17 |
| Campora | 3 |
| Camposano | 32 |
| Cancello e Arnone | 19 |
| Candida | 15 |
| Cannalonga | 9 |
| Capaccio | 14 |
| Capodrise | 35 |
| Caposele | 13 |
| Capri | 28 |
| Capriati a Volturno | 8 |
| Capriglia Irpina | 19 |
| Capua | 26 |
| Carbonara di Nola | 22 |
| Cardito | 41 |
| Carife | 12 |
| Carinaro | 39 |
| Carinola | 18 |
| Casagiove | 23 |
| Casal di Principe | 24 |
| Casalbore | 12 |
| Casalbuono | 2 |
| Casalduni | 42 |
| Casaletto Spartano | 13 |
| Casalnuovo di Napoli | 47 |
| Casaluce | 20 |
| Casalvelino | 10 |
| Casamarciano | 25 |
| Casamicciola Terme | 6 |
| Casandrino | 43 |
| Casapesenna | 22 |
| Casapulla | 29 |
| Casavatore | 43 |
| Caselle in Pittari | 5 |
| Caserta | 29 |
| Casola di Napoli | 16 |
| Casoria | 58 |
| Cassano Irpino | 3 |
| Castel Baronia | 10 |
| Castel Campagnano | 10 |
| Castel di Sasso | 9 |
| Castel Morrone | 7 |
| Castel San Giorgio | 19 |
| Castel San Lorenzo | 9 |
| Castelcivita | 8 |
| Castelfranci | 8 |
| Castelfranco in Miscano | 24 |
| Castellabate | 17 |
| Castellammare di Stabia | 45 |
| Castello di Cisterna | 50 |
| Castello Matese | 9 |
| Castelnuovo Cilento | 7 |
| Castelnuovo di Conza | 19 |
| Castelpagano | 15 |
| Castelpoto | 18 |
| Castelvenere | 10 |
| Castelvetere in Valfortore | 24 |
| Castelvetere sul Calore | 5 |
| Castelvolturno | 44 |
| Castiglione del Genovesi | 7 |
| Cautano | 16 |
| Cava dei Tirreni | 35 |
| Celle di Bulgheria | 12 |
| Cellole | 20 |
| Centola | 11 |
| Ceppaloni | 7 |
| Ceraso | 4 |
| Cercola | 34 |
| Cerreto Sannita | 6 |
| Cervinara | 18 |
| Cervino | 16 |
| Cesa | 26 |
| Cesinali | 23 |
| Cetara | 7 |
| Chianche | 8 |
| Chiusano San Domenico | 9 |
| Cicciano | 30 |
| Cicerale | 10 |
| Cimitile | 35 |
| Ciorlano | 13 |
| Circello | 17 |
| Colle Sannita | 17 |
| Colliano | 11 |
| Comiziano | 26 |
| Conca dei Marini | 22 |
| Conca della Campania | 7 |
| Contrada | 16 |
| Controne | 11 |
| Contursi Terme | 15 |
| Conza della Campania | 17 |
| Corbara | 10 |
| Corleto Monforte | 9 |
| Crispano | 47 |
| Cuccaro Vetere | 1 |
| Curti | 24 |
| Cusano Mutri | 14 |
| Domicella | 19 |
| Dragoni | 13 |
| Dugenta | 12 |
| Durazzano | 16 |
| Eboli | 24 |
| Ercolano | 34 |
| Faicchio | 11 |
| Falciano del Massico | 16 |
| Felitto | 5 |
| Fisciano | 30 |
| Flumeri | 21 |
| Foglianise | 11 |
| Foiano di Val Fortore | 23 |
| Fontanarosa | 14 |
| Fontegreca | 7 |
| Forchia | 10 |
| Forino | 17 |
| Forio | 9 |
| Formicola | 7 |
| Fragneto l'Abate | 9 |
| Fragneto Monforte | 13 |
| Francolise | 19 |
| Frasso Telesino | 9 |
| Frattamaggiore | 56 |
| Frattaminore | 40 |
| Frigento | 26 |
| Frignano | 22 |
| Furore | 19 |
| Futani | 8 |
| Gallo Matese | 0 |
| Galluccio | 12 |
| Gesualdo | 12 |
| Giano Vetusto | 7 |
| Giffoni Sei Casali | 9 |
| Giffoni Valle Piana | 21 |
| Ginestra degli Schiavoni | 23 |
| Gioi | 7 |
| Gioia Sannitica | 18 |
| Giugliano in Campania | 65 |
| Giungano | 14 |
| Gragnano | 17 |
| Grazzanise | 27 |
| Greci | 12 |
| Gricignano d'Aversa | 36 |
| Grottaminarda | 11 |
| Grottolella | 18 |
| Grumo Nevano | 44 |
| Guardia Lombardi | 9 |
| Guardia Sanframondi | 11 |
| Ischia | 10 |
| Ispani | 5 |
| Lacco Ameno | 12 |
| Lacedonia | 17 |
| Lapio | 11 |
| Laureana Cilento | 9 |
| Laurino | 6 |
| Laurito | 0 |
| Lauro | 13 |
| Laviano | 7 |
| Letino | 9 |
| Lettere | 11 |
| Liberi | 17 |
| Limatola | 12 |
| Lioni | 10 |
| Liveri | 24 |
| Luogosano | 28 |
| Lusciano | 27 |
| Lustra | 4 |
| Macerata Campania | 23 |
| Maddaloni | 37 |
| Magliano Vetere | 7 |
| Maiori | 2 |
| Manocalzati | 27 |
| Marano di Napoli | 26 |
| Marcianise | 48 |
| Mariglianella | 30 |
| Marigliano | 38 |
| Marzano Appio | 10 |
| Marzano di Nola | 21 |
| Massa di Somma | 22 |
| Massa Lubrense | 14 |
| Melito di Napoli | 51 |
| Melito Irpino | 10 |
| Melizzano | 14 |
| Mercato Sanseverino | 33 |
| Mercogliano | 15 |
| Meta di Sorrento | 22 |
| Mignano Monte Lungo | 8 |
| Minori | 8 |
| Mirabella Eclano | 13 |
| Moiano | 10 |
| Moio della Civitella | 4 |
| Molinara | 17 |
| Mondragone | 21 |
| Montaguto | 8 |
| Montano Antilia | 3 |
| Monte di Procida | 26 |
| Monte San Giacomo | 14 |
| Montecalvo Irpino | 11 |
| Montecorice | 15 |
| Montecorvino Pugliano | 35 |
| Montecorvino Rovella | 7 |
| Montefalcione | 12 |
| Montefalcone di Val Fortore | 20 |
| Monteforte Cilento | 5 |
| Monteforte Irpino | 23 |
| Montefredane | 32 |
| Montefusco | 11 |
| Montella | 12 |
| Montemarano | 6 |
| Montemiletto | 15 |
| Montesano sulla Maricani | 6 |
| Montesarchio | 25 |
| Monteverde | 12 |
| Montoro | 20 |
| Morcone | 12 |
| Morigerati | 2 |
| Morra de Sanctis | 17 |
| Moschiano | 13 |
| Mugnano del Cardinale | 10 |
| Mugnano di Napoli | 36 |
| Naples | 100 |
| Nocera Inferiore | 31 |
| Nocera Superiore | 30 |
| Nola | 51 |
| Novi Velia | 8 |
| Nusco | 25 |
| Ogliastro Cilento | 18 |
| Olevano sul Tusciano | 6 |
| Oliveto Citra | 20 |
| Omignano | 3 |
| Orria | 1 |
| Orta d'Atella | 36 |
| Ospedaletto d'Alpinolo | 18 |
| Ottati | 2 |
| Ottaviano | 25 |
| Padula | 11 |
| Paduli | 18 |
| Pagani | 39 |
| Pago del Vallo di Lauro | 17 |
| Pago Veiano | 14 |
| Palma Campania | 30 |
| Palomonte | 21 |
| Pannarano | 19 |
| Paolisi | 9 |
| Parete | 26 |
| Parolise | 23 |
| Pastorano | 34 |
| Paternopoli | 13 |
| Paupisi | 16 |
| Pellezzano | 13 |
| Perdifumo | 6 |
| Perito | 5 |
| Pertosa | 9 |
| Pesco Sannita | 10 |
| Petina | 8 |
| Petruro Irpino | 9 |
| Piaggine | 5 |
| Piana di Monte Verna | 18 |
| Piano di Sorrento | 18 |
| Piedimonte Matese | 8 |
| Pietradefusi | 13 |
| Pietramelara | 12 |
| Pietraroja | 13 |
| Pietrastornina | 6 |
| Pietravairano | 11 |
| Pietrelcina | 21 |
| Pignataro Maggiore | 29 |
| Pimonte | 8 |
| Pisciotta | 4 |
| Poggiomarino | 33 |
| Polla | 14 |
| Pollena Trocchia | 27 |
| Pollica | 5 |
| Pomigliano d'Arco | 53 |
| Pompei | 33 |
| Ponte | 19 |
| Pontecagnano Faiano | 36 |
| Pontelandolfo | 13 |
| Pontelatone | 13 |
| Portici | 51 |
| Portico di Caserta | 30 |
| Positano | 11 |
| Postiglione | 20 |
| Pozzuoli | 39 |
| Praiano | 16 |
| Prata di Principato Ultra | 21 |
| Prata Sannita | 7 |
| Pratella | 9 |
| Pratola Serra | 27 |
| Presenzano | 17 |
| Prignano Cilento | 12 |
| Procida | 23 |
| Puglianello | 20 |
| Quadrelle | 6 |
| Qualiano | 39 |
| Quarto | 25 |
| Quindici | 18 |
| Ravello | 11 |
| Raviscanina | 7 |
| Recale | 33 |
| Reino | 14 |
| Riardo | 21 |
| Ricigliano | 8 |
| Rocca d'Evandro | 10 |
| Rocca San Felice | 14 |
| Roccabascerana | 10 |
| Roccadaspide | 10 |
| Roccagloriosa | 15 |
| Roccamonfina | 7 |
| Roccapiemonte | 23 |
| Roccarainola | 17 |
| Roccaromana | 9 |
| Rocchetta e Croce | 8 |
| Rofrano | 1 |
| Romagnano al Monte | 5 |
| Roscigno | 10 |
| Rotondi | 12 |
| Rutino | 10 |
| Ruviano | 13 |
| Sacco | 8 |
| Sala Consilina | 16 |
| Salento | 7 |
| Salerno | 33 |
| Salvitelle | 7 |
| Salza Irpina | 7 |
| San Bartolomeo in Galdo | 23 |
| San Cipriano d'Aversa | 22 |
| San Cipriano Picentino | 14 |
| San Felice a Cancello | 21 |
| San Gennaro Vesuviano | 34 |
| San Giorgio a Cremano | 51 |
| San Giorgio del Sannio | 21 |
| San Giorgio la Molara | 18 |
| San Giovanni a Piro | 7 |
| San Giuseppe Vesuviano | 35 |
| San Gregorio Magno | 9 |
| San Gregorio Matese | 8 |
| San Leucio del Sannio | 11 |
| San Lorenzello | 10 |
| San Lorenzo Maggiore | 17 |
| San Lupo | 10 |
| San Mango Piemonte | 13 |
| San Mango sul Calore | 11 |
| San Marcellino | 30 |
| San Marco dei Cavoti | 26 |
| San Marco Evangelista | 36 |
| San Martino Sannita | 7 |
| San Martino Valle Caudina | 14 |
| San Marzano sul Sarno | 30 |
| San Mauro Cilento | 5 |
| San Mauro la Bruca | 7 |
| San Michele di Serino | 17 |
| San Nazzaro | 12 |
| San Nicola Baronia | 7 |
| San Nicola la Strada | 50 |
| San Nicola Manfredi | 11 |
| San Paolo Bel Sito | 24 |
| San Pietro al Tanagro | 18 |
| San Pietro Infine | 10 |
| San Potito Sannitico | 8 |
| San Potito Ultra | 18 |
| San Prisco | 19 |
| San Rufo | 6 |
| San Salvatore Telesino | 10 |
| San Sebastiano al Vesuvio | 34 |
| San Sossio Baronia | 10 |
| San Tammaro | 20 |
| San Valentino Torio | 34 |
| San Vitaliano | 28 |
| Santa Croce del Sannio | 10 |
| Santa Lucia di Serino | 9 |
| Santa Maria a Vico | 18 |
| Santa Maria Capua Vetere | 35 |
| Santa Maria La Carità | 30 |
| Santa Maria la Fossa | 18 |
| Santa Marina | 11 |
| Santa Paolina | 11 |
| Sant'Agata dei Goti | 20 |
| Sant'Agnello | 25 |
| Sant'Anastasia | 23 |
| Sant'Andrea di Conza | 16 |
| Sant'Angelo a Cupolo | 11 |
| Sant'Angelo a Fasanella | 5 |
| Sant'Angelo a Scala | 9 |
| Sant'Angelo all'Esca | 20 |
| Sant'Angelo d'Alife | 11 |
| Sant'Angelo dei Lombardi | 16 |
| Sant'Antimo | 33 |
| Sant'Antonio Abate | 39 |
| Sant'Arcangelo Trimonte | 16 |
| Sant'Arpino | 25 |
| Sant'Arsenio | 12 |
| Sant'Egidio del Monte Albino | 36 |
| Santo Stefano del Sole | 10 |
| Santomenna | 20 |
| Sanza | 15 |
| Sapri | 13 |
| Sarno | 37 |
| Sassano | 12 |
| Sassinoro | 9 |
| Saviano | 27 |
| Savignano Irpino | 21 |
| Scafati | 42 |
| Scala | 12 |
| Scampitella | 9 |
| Scisciano | 25 |
| Senerchia | 10 |
| Serino | 12 |
| Serramezzana | 4 |
| Serrara Fontana | 4 |
| Serre | 19 |
| Sessa Aurunca | 18 |
| Sessa Cilento | 9 |
| Siano | 16 |
| Sicignano degli Alburni | 15 |
| Sirignano | 8 |
| Solofra | 15 |
| Solopaca | 18 |
| Somma Vesuviana | 26 |
| Sorbo Serpico | 10 |
| Sorrento | 21 |
| Sparanise | 29 |
| Sperone | 23 |
| Stella Cilento | 8 |
| Stio | 9 |
| Striano | 29 |
| Sturno | 9 |
| Succivo | 33 |
| Summonte | 13 |
| Taurano | 14 |
| Taurasi | 18 |
| Teano | 25 |
| Teggiano | 24 |
| Telese Terme | 16 |
| Teora | 13 |
| Terzigno | 33 |
| Teverola | 43 |
| Tocco Caudio | 11 |
| Tora e Piccilli | 13 |
| Torchiara | 8 |
| Torella dei Lombardi | 20 |
| Torraca | 9 |
| Torre Annunziata | 66 |
| Torre Del Greco | 39 |
| Torre le Nocelle | 18 |
| Torre Orsaia | 12 |
| Torrecuso | 30 |
| Torrioni | 7 |
| Tortorella | 13 |
| Tramonti | 7 |
| Trecase | 26 |
| Trentinara | 9 |
| Trentola Ducenta | 28 |
| Trevico | 6 |
| Tufino | 28 |
| Tufo | 11 |
| Vairano Patenora | 11 |
| Vallata | 12 |
| Valle Agricola | 8 |
| Valle dell'Angelo | 7 |
| Valle di Maddaloni | 10 |
| Vallesaccarda | 11 |
| Vallo della Lucania | 14 |
| Valva | 7 |
| Venticano | 19 |
| Vibonati | 2 |
| Vico Equense | 11 |
| Vietri sul Mare | 12 |
| Villa di Briano | 22 |
| Villa Literno | 91 |
| Villamaina | 18 |
| Villanova del Battista | 8 |
| Villaricca | 32 |
| Visciano | 21 |
| Vitulano | 16 |
| Vitulazio | 28 |
| Volla | 58 |
| Volturara Irpina | 9 |
| Zungoli | 12 |

Supplementary Table 1. Municipal pressure indices of all municipalities in the Campania Region

| Municipality | Residents by municipality (2011 Census) | Cluster | MPI | Synthetic index weighted for resident population applied to cluster | Resident population in cluster (2011 Census) |
| --- | --- | --- | --- | --- | --- |
| HIGH IMPACT | | | | | |
| Naples | 985,450 | high 12 | 100 | 98.8 | 1,055,325 |
| Villa Literno | 11,323 | high 12 | 91 |  |  |
| Acerra | 58,552 | high 12 | 81 |  |  |
| Caivano | 38,315 | high 11 | 65 | 64.6 | 185,372 |
| Giugliano in Campania | 110,858 | high 11 | 65 |  |  |
| Arzano | 36,199 | high 11 | 63 |  |  |
| Casoria | 80,425 | high 10 | 58 | 55.5 | 240,476 |
| Volla | 23,276 | high 10 | 58 |  |  |
| Frattamaggiore | 30,758 | high 10 | 56 |  |  |
| Afragola | 65,907 | high 10 | 53 |  |  |
| Pomigliano d'Arco | 40,110 | high 10 | 53 |  |  |
| Melito di Napoli | 38,348 | high 9 | 51 | 49.6 | 306,075 |
| Nola | 33,969 | high 9 | 51 |  |  |
| Portici | 56,856 | high 9 | 51 |  |  |
| San Giorgio a Cremano | 45,920 | high 9 | 51 |  |  |
| Castello di Cisterna | 7,480 | high 9 | 50 |  |  |
| San Nicola la Strada | 21,011 | high 9 | 50 |  |  |
| Marcianise | 40,071 | high 9 | 48 |  |  |
| Casalnuovo di Napoli | 50,055 | high 9 | 47 |  |  |
| Crispano | 12,365 | high 9 | 47 |  |  |
| Castel Volturno | 23,068 | high 8 | 44 | 42.9 | 109,316 |
| Grumo Nevano | 18,029 | high 8 | 44 |  |  |
| Casandrino | 13,208 | high 8 | 43 |  |  |
| Casavatore | 18,590 | high 8 | 43 |  |  |
| Teverola | 13,549 | high 8 | 43 |  |  |
| Cardito | 22,872 | high 8 | 41 |  |  |
| Frattaminore | 16,452 | high 7 | 40 | 38.5 | 340,131 |
| Carinaro | 6,817 | high 7 | 39 |  |  |
| Pozzuoli | 80,987 | high 7 | 39 |  |  |
| Qualiano | 24,695 | high 7 | 39 |  |  |
| Torre del Greco | 88,121 | high 7 | 39 |  |  |
| Aversa | 53,324 | high 7 | 38 |  |  |
| Marigliano | 30,162 | high 7 | 38 |  |  |
| Maddaloni | 39,573 | high 7 | 37 |  |  |
| Boscoreale | 28,730 | high 6 | 36 | 34.5 | 370,977 |
| Gricignano di Aversa | 10,483 | high 6 | 36 |  |  |
| Mugnano di Napoli | 34,768 | high 6 | 36 |  |  |
| Orta di Atella | 24,880 | high 6 | 36 |  |  |
| San Marco Evangelista | 6,669 | high 6 | 36 |  |  |
| Capodrise | 9,647 | high 6 | 35 |  |  |
| Cimitile | 7,037 | high 6 | 35 |  |  |
| San Giuseppe Vesuviano | 27,699 | high 6 | 35 |  |  |
| Santa Maria Capua Vetere | 32,934 | high 6 | 35 |  |  |
| Cercola | 18,141 | high 6 | 34 |  |  |
| Ercolano | 57,078 | high 6 | 34 |  |  |
| Pastorano | 2,898 | high 6 | 34 |  |  |
| San Gennaro Vesuviano | 10,983 | high 6 | 34 |  |  |
| San Sebastiano al Vesuvio | 9,145 | high 6 | 34 |  |  |
| Poggiomarino | 22,095 | high 6 | 33 |  |  |
| Recale | 7,623 | high 6 | 33 |  |  |
| Sant'Antimo | 34,736 | high 6 | 33 |  |  |
| Succivo | 8,061 | high 6 | 33 |  |  |
| Terzigno | 17,370 | high 6 | 33 |  |  |
| Camposano | 5,297 | high 5 | 32 | 30.8 | 108,083 |
| Villaricca | 31,099 | high 5 | 32 |  |  |
| Brusciano | 16,202 | high 5 | 31 |  |  |
| Cicciano | 12,743 | high 5 | 30 |  |  |
| Mariglianella | 7,505 | high 5 | 30 |  |  |
| Palma Campania | 14,906 | high 5 | 30 |  |  |
| Portico di Caserta | 7,650 | high 5 | 30 |  |  |
| San Marcellino | 12,681 | high 5 | 30 |  |  |
| Casapulla | 8,180 | high 4 | 29 | 28.2 | 192,448 |
| Caserta | 76,819 | high 4 | 29 |  |  |
| Pignataro Maggiore | 6,193 | high 4 | 29 |  |  |
| Sparanise | 7,719 | high 4 | 29 |  |  |
| Striano | 8,405 | high 4 | 29 |  |  |
| San Vitaliano | 6,321 | high 4 | 28 |  |  |
| Trentola Ducenta | 17,656 | high 4 | 28 |  |  |
| Tufino | 3,758 | high 4 | 28 |  |  |
| Vitulazio | 6,919 | high 4 | 28 |  |  |
| Grazzanise | 7,082 | high 4 | 27 |  |  |
| Lusciano | 14,406 | high 4 | 27 |  |  |
| Pollena Trocchia | 13,388 | high 4 | 27 |  |  |
| Saviano | 15,602 | high 4 | 27 |  |  |
| Calvizzano | 12,703 | high 3 | 26 | 25.3 | 287,442 |
| Capua | 20,468 | high 3 | 26 |  |  |
| Cesa | 8,460 | high 3 | 26 |  |  |
| Comiziano | 1,817 | high 3 | 26 |  |  |
| Marano di Napoli | 57,673 | high 3 | 26 |  |  |
| Parete | 10,956 | high 3 | 26 |  |  |
| Somma Vesuviana | 36,037 | high 3 | 26 |  |  |
| Trecase | 9,010 | high 3 | 26 |  |  |
| Casamarciano | 3,266 | high 3 | 25 |  |  |
| Ottaviano | 23,947 | high 3 | 25 |  |  |
| Quarto | 39,952 | high 3 | 25 |  |  |
| Sant'Arpino | 13,967 | high 3 | 25 |  |  |
| Scisciano | 5,757 | high 3 | 25 |  |  |
| Boscotrecase | 10,547 | high 3 | 24 |  |  |
| Casal di Principe | 20,844 | high 3 | 24 |  |  |
| Curti | 7,002 | high 3 | 24 |  |  |
| Liveri | 1,637 | high 3 | 24 |  |  |
| San Paolo Bel Sito | 3,399 | high 3 | 24 |  |  |
| Casagiove | 13,548 | high 2 | 23 | 21.8 | 159,832 |
| Macerata Campania | 10,533 | high 2 | 23 |  |  |
| Sant'Anastasia | 28,105 | high 2 | 23 |  |  |
| Carbonara di Nola | 2,269 | high 2 | 22 |  |  |
| Casapesenna | 6,576 | high 2 | 22 |  |  |
| Frignano | 9,219 | high 2 | 22 |  |  |
| Massa di Somma | 5,559 | high 2 | 22 |  |  |
| San Cipriano d'Aversa | 13,398 | high 2 | 22 |  |  |
| Villa di Briano | 6,008 | high 2 | 22 |  |  |
| Mondragone | 27,935 | high 2 | 21 |  |  |
| San Felice a Cancello | 17,189 | high 2 | 21 |  |  |
| Visciano | 4,523 | high 2 | 21 |  |  |
| Casaluce | 9,985 | high 2 | 20 |  |  |
| San Tammaro | 4,985 | high 2 | 20 |  |  |
| Cancello e Arnone | 5,418 | high 1 | 19 | 17.9 | 49,579 |
| Francolise | 4,847 | high 1 | 19 |  |  |
| San Prisco | 11,976 | high 1 | 19 |  |  |
| Carinola | 8,011 | high 1 | 18 |  |  |
| Santa Maria la Fossa | 2,677 | high 1 | 18 |  |  |
| Roccarainola | 7,098 | high 1 | 17 |  |  |
| Calvi Risorta | 5,685 | high 1 | 16 |  |  |
| Falciano del Massico | 3,867 | high 1 | 16 |  |  |
| MEDIUM IMPACT | | | | | |
| Torre Annunziata | 44,780 | medium 3 | 66 | 45.3 | 291,239 |
| Castellammare di Stabia | 67,186 |  | 45 |  |  |
| Angri | 33,477 |  | 44 |  |  |
| Scafati | 50,096 |  | 42 |  |  |
| Pagani | 34,992 |  | 39 |  |  |
| Sant'Antonio Abate | 19,367 |  | 39 |  |  |
| Sarno | 32,732 |  | 37 |  |  |
| Sant'Egidio del Monte Albino | 8,609 |  | 36 |  |  |
| San Valentino Torio | 10,359 | medium 2 | 34 | 31.3 | 166,004 |
| Mercato San Severino | 21,817 |  | 33 |  |  |
| Pompei | 26,190 |  | 33 |  |  |
| Nocera Inferiore | 46,626 |  | 31 |  |  |
| Fisciano | 14,014 |  | 30 |  |  |
| Nocera Superiore | 24,826 |  | 30 |  |  |
| San Marzano sul Sarno | 10,110 |  | 30 |  |  |
| Santa Maria la Carità | 12,062 |  | 30 |  |  |
| Roccapiemonte | 9,002 | medium 1 | 23 | 18.5 | 65,647 |
| Montoro | 19,357 |  | 20 |  |  |
| Castel San Giorgio | 13,270 |  | 19 |  |  |
| Calvanico | 1,671 |  | 16 |  |  |
| Siano | 9,927 |  | 16 |  |  |
| Solofra | 12,420 |  | 15 |  |  |
| Avellino | 54,366 | Valle del Sabato | 40 | 35.5 | 81,205 |
| Montefredane | 2,273 |  | 32 |  |  |
| Atripalda | 10,888 |  | 28 |  |  |
| Manocalzati | 3,184 |  | 27 |  |  |
| Pratola Serra | 3,620 |  | 27 |  |  |
| Aiello del Sabato | 3,928 |  | 22 |  |  |
| Prata di Principato Ultra | 2,946 |  | 21 |  |  |
| Salerno | 133,811 | Valle dell’Irno Cluster 2 | 33 | 33 | 133,811 |
| Baronissi | 16,565 | Valle dell’Irno Cluster 2 | 23 | 19.0 | 27,607 |
| Pellezzano | 11,042 |  | 13 |  |  |
| LOW IMPACT | | | | | |
| Palomonte | 3,904 | low 3 | 21 | 17.5 | 34,885 |
| Oliveto Citra | 3,702 | low 3 | 20 |  |  |
| Santomenna | 414 | low 3 | 20 |  |  |
| Castelnuovo di Conza | 602 | low 3 | 19 |  |  |
| Buccino | 4,778 | low 3 | 18 |  |  |
| Ascea | 5,949 | low 3 | 17 |  |  |
| Castellabate | 8,956 | low 3 | 17 |  |  |
| Contursi Terme | 3,312 | low 3 | 15 |  |  |
| Sicignano degli Alburni | 3,268 | low 3 | 15 |  |  |
| Montecorice | 2,631 | low 2 | 15 | 11.1 | 24,352 |
| Caposele | 3,370 | low 2 | 13 |  |  |
| Centola | 5,087 | low 2 | 11 |  |  |
| Colliano | 3,521 | low 2 | 11 |  |  |
| Casalvelino | 5,468 | low 2 | 10 |  |  |
| Senerchia | 770 | low 2 | 10 |  |  |
| Calabritto | 2,314 | low 2 | 9 |  |  |
| Laureana Cilento | 1,191 | low 2 | 9 |  |  |
| San Gregorio Magno | 4,089 | low 1 | 9 | 6.6 | 17,190 |
| Ricigliano | 1,102 | low 1 | 8 |  |  |
| Stella Cilento | 689 | low 1 | 8 |  |  |
| Laviano | 1,378 | low 1 | 7 |  |  |
| San Mauro la Bruca | 549 | low 1 | 7 |  |  |
| Valva | 1,617 | low 1 | 7 |  |  |
| Perdifumo | 1,767 | low 1 | 6 |  |  |
| Pollica | 2,284 | low 1 | 5 |  |  |
| San Mauro Cilento | 868 | low 1 | 5 |  |  |
| Pisciotta | 2,555 | low 1 | 4 |  |  |
| Serramezzana | 292 | low 1 | 4 |  |  |

Supplementary table 2. Identification of Clusters within the Impact Areas applicable to the geo-stratified recruitment plan as part of a monitoring study in the Campania Region
